# Books Received for Review

**Published:** 1861-10

**Authors:** 


*A treatise on Disease of the Joints. By* Richard Barwell F. R. C. S., *Assistant Surgeon Charing Cross Hospital, etc, Illustrated by engravings on wood. Philadelphia:* Blanchard & Lea.

This is a treatise on diseases of the joints equal to, or rather beyond, the current knowledge of the day, and supplies a deficiency which has long been required. We do not know how better to impress upon our readers the value and importance of this work than to give the abstract of the table of contents, with the assurance that the subjects treated, are most ably and satisfactorily disposed of, nothing is left unfinished.

The chapters which have particularly interested us are—Chapter 1, *Physiological Anatomy of the Joints;* Chapter iii, *Acute Rheumatism;* Chapter iv, *Pyarthrosis.* Poisoning from purulent absorption, as it has been denominated, is fully discussed, and constitutes a most interesting chapter, of great practical importance. Chapter xiv, *High Joint Disease,* is also full of suggestion, and instruction ; Chapter xviii, *On Removal of Diseased Joints,* containing full description of circumstances which justify removal of a diseased joint, with causes of preference, for amputation or excision, and directions upon some important points generally to be observed, in the operation for excision of points ; also, a description of the reparitive process after excission, and special directions for operation upon the different joints.

This last chapter is of the greatest value to the surgeon, and every one who proposes to operate upon diseased joints, for their removal will do well to consider what has been so well written in this chapter.

The great frequency of diseases of the joints, and the importance of thoroughly understanding their nature, and most judicious management will induce physicians to read this most complete treatise upon the whole subject of diseased joints.

The chapters not mentioned, are all of equal interest and importance ; they are omitted in this notice for want of room for a more lengthy description.

*Report of an Inquest held on the body of* Mary Gearon, *of Fort Erie, Canada West, before the Coroner of the County,* Dr. Kempson, *being an exact reprint of the Coroner's notes when at the inquest.*

This inquest was held upon the body of a woman who died in labor. The child was turned and *delivered* except the head. The vagina was lacerated, and the head found in the cavity of the abdomen. There was also some injury to a portion of the jejunum. Laceration of the vagina was sufficient to cause death, in the opinion of the physicians who gave testimony. The case was a very remarkable one, and the physicians who had it in care must prepare the copy, if they desire to have it reported. The medical testimony is of interest and importance in various respects, and probably on this account it has been published for the purpose of more general circulation. We do not propose to review either the practice of the physicians or the testimony at the inquest, but follow the example of our ancient historian Josephus, who, after giving an account of some of the miracles, would "let every onethink of these matters as he pleaseth."

